# The role of morphemic knowledge during novel word learning

**DOI:** 10.1177/17470218231216369

**Published:** 2023-12-07

**Authors:** Ali Behzadnia, Johannes C. Ziegler, Danielle Colenbrander, Audrey Bürki, Elisabeth Beyersmann

**Affiliations:** 1 Department of Linguistics, University of Potsdam, Potsdam, Germany; 2 International Doctorate for Experimental Approaches to Language and Brain (IDEALAB), University of Potsdam (DE), University of Groningen (NL), University of Newcastle (UK), and Macquarie University (AU); 3 School of Psychological Sciences, Macquarie University, Sydney, NSW, Australia; 4 Centre for Reading, Macquarie University, Sydney, NSW, Australia; 5 Laboratoire de Psychologie Cognitive, Centre National de la Recherche Scientifique, Aix-Marseille Université, Marseille, France; 6 Australian Centre for the Advancement of Literacy (ACAL), Faculty of Education and Arts, Australian Catholic University, Sydney, NSW, Australia

**Keywords:** Morphological family size, morphological structure, novel word acquisition, written training

## Abstract

This study used a novel word learning paradigm to investigate the role of morphology in the acquisition of complex words, when participants have no prior lexical knowledge of the embedded morphemic constituents. The influence of morphological family size on novel word learning was examined by comparing novel stems (*torb*) combined with large morphological families (e.g., *torbnel*, *torbilm*, *torbla*, *torbiph*) as opposed to small morphological families (e.g., *torbilm*, *torbla*). In two online experiments, participants learned complex novel words by associating words with pictures. Following training, participants performed a recognition and a spelling task where they were exposed to novel words that either did or did not contain a trained morpheme. As predicted, items consisting of a trained and an untrained constituent were harder to reject but easier to spell than those that did not contain any trained constituents. Moreover, novel words including trained constituents with large morphological families were harder to reject than those including constituents with small morphological families. The findings suggest that participants acquired novel morphemic constituents without prior knowledge of the constituents and point to the important facilitatory role of morphological family size in novel word learning.

## Introduction

Morphemes represent the smallest units of meaning within a word and have been shown to play an important role in the acquisition of new vocabulary (e.g., Rastle & Taylor, 2018). Free morphemes like *car* and *farm* can stand alone as single words, whereas bound morphemes like affixes (e.g., -*er, -ing*) can only appear in combination with free morphemes (e.g., *farmer*). In English, the majority of words are morphologically complex, that is, they consist of multiple morphemic units (e.g., *teach* *+* *er: teacher; un* *+* *fair: unfair; text* *+* *book: textbook*). Much research has demonstrated that skilled readers automatically segment morphologically complex words into their constituent morphemes during reading tasks (e.g., Beyersmann et al., 2016; Diependaele et al., 2009; Rastle et al., 2004; Taft & Nguyen-Hoan, 2010). Knowledge of constituent morphemes also plays a key role in language comprehension and is, as we discuss below, particularly important for understanding new (or unknown) words formed by combinations of embedded morphemes. Morphological segmentation can be used as a tool to derive meaning from new words (e.g., *anti-mask-er* = “a person who resists wearing a mask”) and therefore has the potential to support vocabulary acquisition. While even skilled readers tend to regularly encounter new words, understanding the mechanisms of complex word acquisition is particularly relevant for individuals who are frequently exposed to novel words in their reading, such as developing readers (Beyersmann, Wegener, Spencer, & Castles, 2022) or those acquiring a second language (Behzadnia et al., 2023). However, little is known regarding if and how readers process morphological structure when being presented with entirely novel letter strings.

The current study had two principal aims. The first aim was to test whether readers acquire embedded morphemic units without the support of any pre-existing lexical knowledge of the constituent morphemes, and then generalise the trained morphemes to an entirely new morphemic context. The second aim was to examine if morphological family size (i.e., the number of morphologically complex words in which a morpheme occurs) influences the learning and recognition of constituent morphemes in adults. Below, we summarise previous studies on the role of morphemic knowledge and morphological family size and discuss the implication for theories of novel word learning.

### Morphemic knowledge in novel word acquisition

A small number of training studies have investigated the role of morphemic knowledge in novel word acquisition. For example, Merkx and colleagues (2011) investigated the role of semantic information on the acquisition of novel sufﬁxes combined with existing stems (e.g., *sleep* + *nept* = *sleepnept*) by directly comparing a form and a semantic-learning condition. In the form-learning condition, participants were exposed to the auditory and written form of each novel word. In the semantic-learning condition, participants were exposed to the written form of the novel words and an auditory presentation of the definition of each novel word. In a recognition and lexical decision task performed after training, participants had more difficulty rejecting novel word items containing an untrained and a trained morpheme than a completely untrained item. Critically, the direction of the training effect in the post-training tasks (i.e., longer response times and higher error rates to novel words consisting of an untrained + trained morpheme compared with an entirely untrained control condition) suggests that items including trained constituents were perceived as more word-like, resulting in greater difficulty in rejecting more “word-like” items. In a definition selection task, also performed after training, participants were asked to select the definitions of trained items and untrained items containing an untrained stem and a trained suffix. The training effect for the untrained items was larger in the semantic than form-learning condition, indicating that participants were able to generalise the meaning of the newly learned suffixes to new words (for converging results, see Tamminen et al., 2015). Further evidence for the generalisation of novel morphemic knowledge was reported by Tamminen and colleagues (2012), who built on Merkx et al.’s (2011) training paradigm. Adult speakers of English were trained on novel words consisting of an existing stem and a novel suffix (i.e., *sleep* + *afe* = *sleepafe*). Testing took place immediately and after 2 days. In a shadowing task (i.e., speeded repetition of spoken novel words) that took place 2 days after training, participants responded faster to and were more accurate in selecting a definition for novel words containing a trained compared with an untrained suffix, thus replicating Merkx et al.’s earlier findings.

Other training studies have investigated the acquisition of novel words containing a novel stem and an existing suffix (e.g., Berko, 1958; Dawson et al., 2021; Tucker et al., 2016). For instance, in Dawson et al.’s (2021) study, participants, in two sessions with a 1-week delay, learned novel words containing a familiar derivational suffix (e.g., *clant* *+* *ist* *=* *clantist*) by associating each with a definition which was either semantically congruent or incongruent with the suffix. The authors reported that in a post-training lexical decision task, novel items containing trained morphemes were harder to reject than completely untrained items (i.e., untrained stem + untrained suffix), hence providing further evidence for the generalisation of trained morphemes to untrained morphemic context.

These previous training studies used combinations of novel and existing morphemes (e.g., *sleep* *+* *nept* or *clant* *+* *ist*). Therefore, it is possible that participants’ familiarity with the existing morphemes contributed to the training effects and generalisation. In other words, although these prior studies support the idea that readers are able to identify a trained embedded morpheme, it is not clear if the observed effect only occurred because acquisition was facilitated by the presence of an already known morpheme. This, of course, does not take away from the importance of the prior findings, because the analysis of novel complex words is often naturally guided by prior knowledge of its morphemic constituents. For instance, a child might acquire the word *light* sooner in their reading development than the word *lighter*, in which case the child’s knowledge of *light* will facilitate the process of morphologically decomposing and deriving meaning from *lighter*. However, the opposite scenario also applies, where readers are exposed to complex novel words without having any knowledge of its embedded morphemic constituents and it is less clear how readers derive meaning from complex words in such a situation. This scenario represents a particularly strong test of how readers identify morphemic boundaries by mapping orthographic input onto meaning, without being able to isolate any embedded morphemic units. In the present study, this point was addressed by using items consisting of two entirely novel morphemes to rule out the possibility that participants would draw on their pre-existing morphological and lexical knowledge.

### Morphological family size and novel word learning

Morphological family size refers to the number of morphologically complex words in which a morpheme occurs. A stem or an affix has a large morphological family size if it is embedded in many morphologically complex words (e.g., *acid* occurs in *acidity*, *acidify*, *acidifier*, and *acidulate*) and a small family size if it is embedded in a few morphologically complex words (e.g., *skull* occurs in *skulls* and *skullcap*). Morphological family size has been shown to be an important predictor of visual word recognition, showing that words with a large morphological family are processed faster and more accurately during lexical decision than words with a small morphological family (e.g., Baayen et al., 1997; Bertram et al., 2000; Beyersmann & Grainger, 2018; Boudelaa & Marslen-Wilson, 2011; De Jong et al., 2002; Juhasz & Berkowitz, 2011; Kuperman et al., 2008; Schreuder & Baayen, 1997); however, the effect of morphological family size on novel word acquisition is less well understood. To the best of our knowledge, only one prior study has reported a facilitatory effect of morphological family size on novel word learning (Tamminen et al., 2015). Similar to Merkx et al. (2011), participants were trained with the forms and definitions of novel words containing an existing stem and a novel suffix (i.e., *sleep* *+* *nept* *=* *sleepnept*), where suffixes differed depending on whether they were part of a large morphological family (e.g., *creepesh*, *grabesh*, *sleepesh*, *sheepesh*), or a small morphological family (e.g., *bringane, lockane*). Following training, participants read aloud sentence final words containing an untrained stem and a trained suffix. Latencies were shorter for the novel words containing an embedded trained suffix with a large family size. Participants also stated whether the meaning of the sentence frame (the words preceding the sentence final word) was semantically congruent with the sentence final word containing an untrained stem and a trained suffix. Response accuracy was higher when the sentence final word contained a trained suffix with a large family size.

These results provide some initial evidence for the idea that the acquisition of morphemic knowledge is facilitated by morphological family size, and converge with the finding that skilled readers process words with a large morphological family faster and more accurately during lexical decision than words with a small morphological family (e.g., Baayen et al., 1997; Bertram et al., 2000; Beyersmann & Grainger, 2018; De Jong et al., 2002; Moscoso del Prado Martin et al., 2004). In the present study, we built on these prior findings to ask if morphological families also support novel word learning in a context where participants have no prior knowledge of the morphemic boundaries between the embedded constituents, as is the case in novel words consisting of two entirely novel constituents. As such, the study’s goal was to test if readers find it easier to detect boundaries between morphemes belonging to a large as opposed to a small morphological family.

### Present study

The present novel word learning study used a series of two online experiments to examine the learning of complex novel words formed by combining novel constituent morphemes (e.g., *torb* + *ilm* = *torbilm*). In this way, prior morphological and lexical knowledge could not be used to guide morphological decomposition and learners had to infer morphological structure solely on the basis of their exposure to different complex novel words. The second experiment served as a replication of the first experiment, using slightly tighter counterbalancing between conditions, while using the exact same design and novel word learning principles. During training, participants had to associate the novel words with pictures of objects. Moreover, we manipulated the morphological family size of the stems. Half of the stems belonged to a large morphological family (i.e., were combined with four different second constituents), whereas the other half belonged to a small morphological family (i.e., were combined with only two different second constituents). Training was repeated until an accuracy threshold of 90% was reached.

Directly following training, participants completed a recognition and spelling task in which their knowledge of the trained constituents was tested, with a third of the items being trained and two-thirds untrained. The primary purpose of these tasks was to test participants’ responses to the untrained items, as a way to investigate their ability to generalise the trained constituents to a new morphemic context, which were subdivided into two key conditions. One condition contained the trained constituents embedded in novel items combined with a second untrained constituent (e.g., *veam* + *elp* = *veamelp*). These were compared against a second condition consisting of two entirely untrained constituents (e.g., *prish* + *ig* = *prishig*). Hence, the analyses of the post-training data were entirely focused on participants’ responses to the untrained trials.

In the recognition task, items were presented individually on a computer screen and participants had to decide if the target was trained or untrained as quickly and accurately as possible. The task was to respond “yes” only to trained items, and to respond “no” to any novel item, even if it contained a trained constituent. We hypothesised that if participants are indeed able to acquire novel morphemes without any pre-existing morphological and lexical knowledge and without ever being exposed to the morphemic units in isolation, this would make it harder to reject items containing a trained embedded constituent as opposed to items not containing a trained constituent. As such, it was expected that familiarity with the trained constituents would have an *inhibitory* effect on responses in the recognition task. We further hypothesised that if morphological family size facilitates learning in a situation where participants cannot benefit from any pre-existing morphological and lexical knowledge during training, this would make it harder to reject items containing embedded constituents with large compared with small morphological families.

In the spelling task, participants were exposed to the spoken forms of each target item and asked to spell it as accurately as possible. It was expected that familiarity with the trained constituents would have a facilitatory effect on responses in the spelling task, because familiarity with the trained constituents would make it easier for participants to spell items containing a trained constituent than an entirely untrained item. We further expected an effect of morphological family size, that is, higher spelling accuracy for items with larger compared with small morphological families.

## Experiment 1

### Method

#### Participants

Fifty native speakers of English (34 females, 16 males, *M*_age_: 30, *SD*: 9.7) participated online for monetary compensation (£7.5/hr). The sample size was established based on the average sum of participants in prior training studies with comparable numbers of novel word items (e.g., Beyersmann et al., 2021; Beyersmann, Wegener, Pescuma, et al., 2022). Participants were recruited via Prolific (www.prolific.co).

All participants were monolingual and raised only with English as their native language. They were born and raised in the United Kingdom and with English as their first and only language. They reported no hearing, vision, and language-related difficulties. Prior to participation, participants were informed about the experimental procedure and written consent was obtained. This study was approved by the ethics committee of Macquarie University, Sydney, Australia.

#### Materials

##### Novel words

Novel first constituents (*n* = 16) and novel second constituents (*n* = 24) were selected and combined to form morphologically complex words (*n* = 48). The first constituents consisted of 4–6 letters, and the second constituents of 2–3 letters. The novel morphemes were orthographically legal and pronounceable letter sequences. The first constituents were selected from a list of English nonwords generated by the ARC nonword database (Rastle et al., 2002). We avoided using orthographically similar novel word stems and checked that none of the stems appeared in the English Lexicon Project (ELP; Balota et al., 2007) and Subtlex-UK (Van Heuven et al., 2014) databases.

The second constituents represented non-morphemic word endings from the ELP-generated list of English words (Balota et al., 2007) to ensure orthographic plausibility of the novel letter strings. The selected word endings did not occur in the MorphoLex database (Sánchez-Gutiérrez et al., 2018), suggesting that they did not have an affixal status or meaning. A native English speaker further confirmed that none of the selected constituents formed existing morphemes of the English language. In addition, the novel words were audio recorded by a native speaker of English.

Meaning was assigned to each of the constituents. The first constituents (e.g., *torb*) always referred to an object (e.g., a ball). The second constituents were used to further qualify the first constituents’ meaning (e.g., *torb* + *ilm* = *big ball*). The online Supplementary Material A shows the complete list of novel words (see also https://osf.io/g827m/). In addition, we extracted concreteness scores from a database by Brysbaert and colleagues (2014) and imageability scores from the Glasgow Norms (Scott et al., 2019) to ensure that the pictures representing the first and second constituents were matched (see Table 1). For concreteness, the rating scale ranged from 1 (*abstract*) to 5 (*concrete*), and for imageability, the rating scale ranged from 1 (*not at all imageable*) to 7 (*highly imageable*).

**Table 1.** Mean item characteristics per word set and constituent morphemes in Experiments 1 and 2.

Word set	Constituent morpheme	Family size	Number of letters	Coltheart’s N	OLD 20	Concreteness	Imageability
Set 1	First constituent	Large	4.75	3.75	1.8	4.93	6.70
		Small	4.75	3.50	1.78	4.94	6.73
	Second constituent	Large	2.87	5.37	1.48	3.51	5.03
		Small	2.75	9.25	1.41	3.10	4.83
Set 2	First constituent	Large	5.00	2.50	1.81	4.93	6.70
		Small	4.75	2.75	1.82	4.94	6.73
	Second constituent	Large	2.75	7.37	1.37	3.51	5.03
		Small	2.75	9.50	1.46	3.10	4.83

For counterbalancing purposes within the current training paradigm, two sets of complex novel words were created with 32 items per set. Half of the participants were trained on Set 1 while Set 2 was used as untrained items in the post-training phase, whereas the other half of participants were trained on Set 2 with Set 1 acting as untrained items in the post-training phase. Each set was further divided into two family size conditions: large family size and small family size. Morphological family size is defined as the number of different morphologically complex words in which a morpheme appears. Relatively small morphological family sizes (i.e., two vs four) were selected to contain the overall number of constituent concatenations, and in turn restrict the overall number of novel words to be learned in this study, thus ensuring feasibility of the training task.

One of the most critical features of the current training paradigm was to carefully control the number of orthographic exposures across training conditions. Each family size condition contained four first constituents. In the large morphological family size condition, each constituent was combined with four different second constituents (*farsherp, farshlor, farshoth, farshib*) and therefore each first constituent appeared four times. In the small family size condition, each constituent was combined with two different second constituents (e.g., *dirchilm, dirchla*), that is, each first constituent occurred only twice. To balance the number of exposures to each first constituent, the novel words in the small family size condition were repeated once (i.e., four exposures to each first constituent), thereby matching the number of exposures in the large family size condition. The consequence of this was that there was an imbalance in the number of exposures to the whole novel words (one exposure in the large family size condition; two exposures in the small family size condition). Although the key to the post-training task was that it assessed participants’ knowledge of the trained constituents, it yet provided an important control for the influence of whole-word exposures onto the here observed learning outcomes.

The novel words’ constituent morphemes were matched across family size conditions on Coltheart’s N (i.e., the number of words that can be generated by a single letter substitution, Coltheart et al., 1977), Orthographic Levenshtein Distance 20 (OLD20, i.e., mean Levenshtein Distance from a word to its 20 closest orthographic neighbours that can be generated by a single letter substitution, deletion, or addition, Yarkoni et al., 2008), and on the number of letters. Coltheart’s N and OLD20 both represent a measure for how related the novel items are to other existing words in the lexicon, which could potentially impact participants’ ability to learn the novel items. Both measures were computed using the “vwr” package (Keuleers, 2013) in the R statistical software (R Core Team, 2020). The mean item characteristics for each condition are reported in Table 1.

##### Pictures

**Figure 1.**
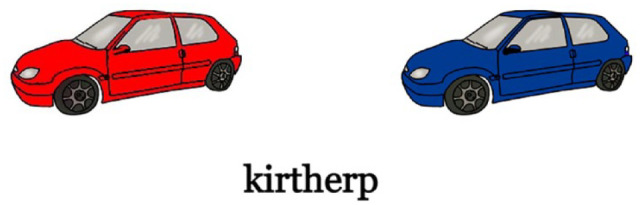
A sample picture used for written novel words training.

Pictures of eight objects were selected from the Multilingual Picture (MultiPic) database (Duñabeitia et al., 2018). Each object picture was associated with one of the first constituents. For example, “kirth” refers to a “car” in these examples: “kirthift,” “kirthiom,” “kirtherp,” and “kirthlor.” We then modified each picture based on the second constituent meanings, for example, cheap, expensive, red, and blue. Second constituent meanings referred to colour (red/blue), size (small/large), price (high/low), age (old/new), or cleanliness (clean/dirty; see Figure 1). It should be noted that the novel words’ meanings in each family size do not form pairs of meanings (i.e., colour, size) and therefore their meanings are independent of one another.

#### Procedure

##### Training phase

**Figure 2.**
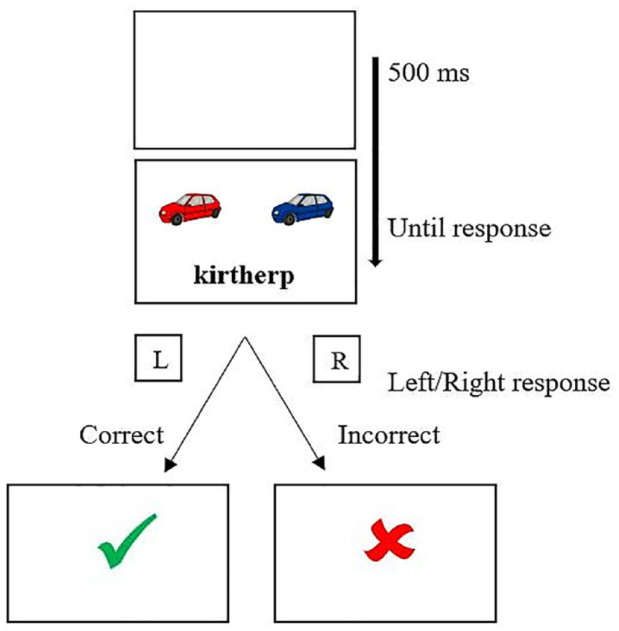
Design of the training phase.

The entire study was designed and implemented online using the Gorilla Experiment Builder (www.gorilla.sc; Anwyl-Irvine et al., 2021). A novel word training paradigm was employed to provide training of morphologically complex novel English words in written form. On each trial, participants were first presented with a blank screen for 500 ms followed by the simultaneous presentation of two pictures of objects and a printed novel word. The latter corresponded to one of the pictures (see Figure 2). The two objects corresponded to the same first constituent meaning but differed in their visual features based on the second constituent meaning. We opted for a task that allowed participants to assign meaning to the embedded reading units. Participants’ familiarity with the meaning of the embedded constituents (e.g., *blue* + *car; red* + *car*) represented an important prerequisite of this task. However, given that the letter strings in this study were entirely novel, participants were unable to draw on any pre-existing lexical knowledge of the embedded morphemic constituents, thus representing a strength of the current experimental training design.

The participants’ task was to associate each novel word with one of the pictures by pressing a keyboard button. Participants were instructed to respond as accurately and quickly as possible and had a maximum of 5,000 ms to do so. Then, they received positive or negative feedback indicating whether or not their response was correct. If they failed to respond within the time limit, they automatically proceeded to the next novel word and pictures without feedback. The order of item presentation was randomised across participants. After the presentation of all novel words and their corresponding pictures, participants received an accuracy percentage score as well as the number of correct and incorrect responses. To complete the training phase, participants had to repeat the task until they reached an accuracy threshold of 90%. The 90% accuracy criterion was calculated based on the entire list of novel words (rather than for individual words). If participants failed to reach the 90% accuracy threshold, they were asked to complete another training run including the entire word list. These procedural settings were adopted to ensure that the number of exposures to items in the small and large family size conditions remained balanced throughout.

##### Reading fluency test

Participants’ reading fluency was measured with a standardised reading fluency test (Test of Word Reading Efficiency [TOWRE]; Torgesen et al., 1999), Form A. The test had two parts. In the first part, participants were required to read lists of English words and in the second part, they read a list of English nonwords. For both lists, stimuli were arranged from easy to more difficult items in terms of the pronunciation and number of syllables. Participants could skip words if they did not know how to read them. This test directly followed the training phase. The online administration of this test followed the same procedure of its in-person administration whereby participants first saw the list of words, and then the list of nonwords, and were instructed to read aloud each item one-by-one in a timely manner. Participants had a maximum of 45 s to complete each list. Voice recordings were used to check for the pronunciation and correct the scoring. It is worth noting that the TOWRE scores are standardised scores rather than raw scores and therefore are not a direct reflection of the number of words/nonwords read correctly. The TOWRE norms are based on a sample of adults. To compute the scores for the word and nonword lists, first we calculated the raw scores which is equal to the number of correctly pronounced items. Then, the raw scores were translated into standard scores. The standard scores for the TOWRE are based on a distribution with a mean of 100 and an *SD* of 15. The mean and standard deviation scores for the lists of words (*M*: 99.50, *SD*: 12.40) and nonwords (*M*: 105.5, *SD*: 8) across all participants were computed. In addition, the scores were computed separately for participants assigned to item Set 1 (words: *M*: 100, *SD*: 13; nonwords: *M*: 106.30, *SD*: 7) and Set 2 (words: *M*: 99, *SD*: 11.60; nonwords: *M*: 105, *SD*: 8.70), showing that participants’ reading proficiency was comparable across participant groups. A mean standard score of ~100 indicated normal reading skills.

##### Post-training phase

This phase included two tasks, a recognition task and a spelling task. Both tasks consisted of three conditions: a trained item condition (e.g., *veamift*), a trained stem condition including a trained first constituent and an untrained second constituent (e.g., *veamelp*), and an untrained stem condition where both constituents were untrained (e.g., *prishig*). Each condition consisted of 24 items. Half of the trained stems belonged to a large and half to a small morphological family. In addition, the presentation of all words was randomised in both tasks.

##### Recognition task

The task started with a presentation of a fixation cross “+” for 500 ms followed by the presentation of a written novel word until response. The task was to decide if the presented item was trained or untrained, as quickly and accurately as possible. Participants had to respond within 4,000 ms using button press responses. They received feedback for the accuracy of their responses. At the end of the task, the scores for the total number of correct and incorrect responses and the mean response accuracy percentage for all the items were provided to each participant.

##### Spelling dictation

A fixation cross “+” was first presented for 500 ms. Subsequently, participants were required to click on a “play” button to listen to the audio recordings for each item. Participants were given the option to listen to each recording up to three times. After each recording, participants were required to type their response in a box appearing on the screen for each item. Spellcheck was disabled and since novel words were used in the experiment it was impossible to rely on online dictionaries or other online tools.

### Analysis

The lme4 package (Bates et al., 2015) was used to run the statistical models in the R statistical software (R Core Team, 2020). We analysed response times and error rates in the recognition task and error rates in the spelling task. For these analyses, the trials were restricted to the untrained conditions. Two different analyses were run for each task and dependent variable. First, to investigate the effect of stem status, we compared responses with novel words containing trained versus untrained stems. The corresponding linear mixed-effects model included stem status as fixed-effect predictor (trained stem condition was coded as 0.5 and untrained stem condition was coded as −0.5). Second, to investigate the effect of morphological family size, we compared responses with trained stems with a large morphological family to trained stems with a small morphological family. The corresponding linear mixed-effects model included morphological family size as predictor (large family size was coded as 0.5 and small family size was coded as −0.5). In all models, the random-effects structure included by-participant and by-item varying intercepts and slopes. The initial model had no correlation between intercepts and slopes. When convergence issues occurred, the model was simplified. When a model has trouble estimating a random term, lmer tends to return a very small value for this random term. Therefore, we removed the random terms one by one, starting with the random term with the smallest value.

The distribution of response times was first visualised with a density plot to detect extreme values. Response times <300 ms and >3,000 ms were considered as outliers and removed. Following Box-Cox tests (Box & Cox, 1964) we used the inverse transformation of response times as the dependent variable. The first converging model was run twice, first on all data points, then following the residual trimming procedure outlined by Baayen and colleagues (2008; Baayen & Milin, 2010) the model was run on all but excluding the data points corresponding to residuals >2.5. Only the results of this second model are reported. To analyse response error rates, we used generalised linear mixed-effects models. The models included response accuracy as the dependent variable (*accuracy* = 1, *error* = 0) and were built in the same way as in the response time analyses. The same statistical procedure was used to analyse the data of Experiment 2. The cut-off and the excluded outliers were different across experiments since the distribution of the response times was different.

### Results and discussion

#### Recognition task

##### Stem status

**Figure 3.**
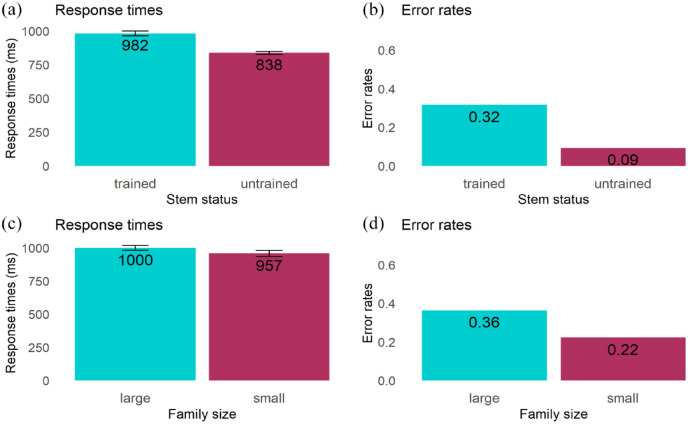
Experiment 1: Effect of stem status and family size in recognition task: (a) Mean response times for the effect of stem status, (b) Mean error rates (%) for the effect of stem status, (c) Mean response times for the effect of family size, and (d) Mean error rates (%) for the effect of family size. *Note.* The standard errors reported in the plots are not corrected for within-subject manipulation for all the plots.

*Response times.* We removed 492 errors (20.5%) out of 2,400 trials from the dataset. Outlier trials were also removed from the dataset (10 out of 1,908 correct responses). The remaining 1,898 trials were included in the analysis. The mean response time was 982 ms (*SD* = 481) in the trained stem condition and 838 ms (*SD* = 349) in the untrained stem condition. The model revealed a significant effect of stem status (β = 1.73 × 10^−4^, *SE* *=* 2.49 × 10^−5^, *t* = 6.98, *p* < .001), indicating slower responses in the trained stem condition than in the untrained stem condition (Figure 3a).

##### Error rates

The model revealed a significant effect of stem status (β = −1.89, *SE* *=* 0.282, *z* = −6.68, *p* < .001), reflecting participants’ less accurate responses in the trained stem condition (*M*: 31.6%, *SD*: 0.64) than in the untrained stem condition (*M*: 9.0%, *SD*: 0.39; Figure 3b).

#### Morphological family size

##### Response times

First, we removed 380 errors (31.6%) out of 1,200 trials from the dataset. Then, outlier trials were also removed (5 out of 820 correct responses). The remaining 815 trials were included in the analysis. The mean response time was 1,000 ms (*SD* = 407) in the large stem family size condition and 957 ms (*SD* = 404) in the small stem family size condition. The statistical model showed no significant effect of family size on response times (β = 6.12 × 10^−5^, *SE* = 3.32 × 10^−5^, *t* = 1.844, *p* = .072; see Figure 3a).

##### Error rates

The statistical model revealed a significant effect of family size (β = −0.837, *SE* = 0.38, *z* = −2.206, *p* = .027), indicating that participants made more errors rejecting items in the large (*M*: 36%, *SD*: 0.567) than in the small family size condition (*M*: 22%, *SD*: 0.498; see Figure 3d).

#### Spelling task

##### Stem status

The model revealed a significant effect of stem status (β = 0.66, *SE* *=* 0.297, *z* = 2.22, *p* = .026), suggesting that participants made fewer errors spelling items containing trained stems (*M*: 35.5%, *SD*: 0.62) than untrained stems (*M*: 47%, *SD*: 0.66).

##### Morphological family size

The mean error rates were 38.3% (*SD* = 0.60) in the large family size condition and 29.5% (*SD* = 0.62) in the small family size condition. The model showed no significant effect of family size on response error (β = −0.577, *SE* *=* 0.452, *z* = −1.276, *p* = .202).

In sum, the results of Experiment 1 are consistent with the hypothesis that native speakers of English are able to identify novel embedded constituent morphemes without any pre-existing morphological and lexical knowledge and without ever encountering the morphemic constituents in isolation. Participants found it harder to reject items consisting of a trained first and an untrained second constituent compared with items consisting of two untrained constituents. This suggests that participants generalised their acquired morphemic knowledge to a new morphemic context. In addition, novel words consisting of an embedded first constituent with a large morphological family and a second untrained constituent were harder to reject as untrained words than those consisting of a first constituent with a small morphological family. The family size effect was present for the analysis of error rates in the recognition task. These results clearly rule out the possibility that constituent learning was facilitated by the larger number of whole-word exposures (one exposure in the large family size condition; two exposures in the small family size condition). Instead, they provide key evidence for the important role of morphological family size in novel word learning, suggesting that morphemic constituents with large morphological families were associated with better learning outcomes than morphemic constituents with small morphological families.^1^

## Experiment 2

While the results of Experiment 1 are straightforward, there were two potential methodological shortcomings that we addressed in Experiment 2. The first point to note is that although the novel letter strings in the two morphological family size conditions of Experiment 1 were closely matched on number of letters, orthographic neighbourhood, and OLD20, the items were never swapped across conditions. As such, it cannot entirely be ruled out that at least some of the differences between conditions may have been due to uncontrolled item specific characteristics. To address this point, the two sets of items from Experiment 1 (Sets 1 and 2; see Supplementary Material A) were split into two further lists (Sets 1a, 1b, 2a, and 2b; see Supplementary Material B), to ensure that every item was assigned to a large morphological family in half of the trials, and to a small morphological family in the other half of the trials. The second potential confound of Experiment 1 was that two-thirds of the post-training trials belonged to the large morphological family size condition, but only one-third to the small family size condition. Is it possible that this bias towards the large morphological family size condition in the post-training trials provided a processing boost for large family size items, rather than reflecting a family size effect that was purely based on the training characteristics themselves. To rule out this potential confound, we decreased the number of post-training trials in the large family condition of Experiment 2. Therefore, there were equal number of trials in both family size conditions.

In line with the outcome of Experiment 1, we hypothesised that if participants identify the morpheme boundaries of novel words and learn them through picture–word associations, there should be a significant embedded stem effect in the post-training tests (i.e., the recognition and spelling tasks). In addition, we hypothesised that stems with large morphological families would be associated with better learning outcomes than stems with small morphological families. We pre-registered our predictions as well as the method, procedure, and the data analysis plan for this second experiment (https://aspredicted.org/blind.php?x=an8hi4).

### Method

#### Participants

A total of 100 native speakers of English (67 females, 33 males, *M*_age_: 25, *SD*: 9.1) were recruited online via Prolific participant pool (www.prolific.co). The sample size of Experiment 2 was calculated in the form of a power analysis based on the data of Experiment 1 (for a full report of the power analysis, see https://osf.io/g827m/). Participants were rewarded £7.5/hr for their participation. All participants were born and raised in the United Kingdom and with English as their first and only language. They were all university students. Participants reported no hearing, vision, and language-related difficulties. Prior to participation, participants were informed about the procedure of the experiment and gave their informed consent. This study was also approved by the ethics committee of [name deleted to maintain the integrity of the review process]. Participants’ reading fluency (Test of Word Reading Efficiency [TOWRE]; Torgesen et al., 1999) scores for the lists of words (*M*: 97, *SD*: 13.60) and nonwords (*M*: 105.5, *SD*: 9) across all participants were computed. In addition, the scores were computed separately for participants assigned to item Set 1 (words: *M*: 97, *SD*: 13.70; nonwords: *M*: 106.30, *SD*: 8.70) and Set 2 (words: *M*: 97.30, *SD*: 13.50; nonwords: *M*: 104.70, *SD*: 8.50), showing that the reading proficiency scores were comparable across the two participant groups.

### Materials

#### Training materials

As in Experiment 1, we used two different sets of items. Within each set, the items were split across two family size conditions, with four novel first constituents per condition. In the large family size condition, each constituent was combined with four different second constituents (i.e., 16 words) and therefore each first constituent was encountered four times. In the small family size condition, each constituent was only combined with two different suffixes (i.e., 8 words), that is, each stem was encountered twice.

To counterbalance items across training conditions, two additional item sets were created by swapping the novel first constituents across family size conditions and within each set, thus leading to four counterbalanced item sets (e.g., large family size Set 1a: *farsherp*, small family size Set 1b: *farshord*, large family size Set 2a: *greachel*, small family size Set 2b: *greachpo*; see Supplementary Material B for a complete list of the trained novel words). Four groups of participants were assigned to each item set during training. During the post-training phase Set 2 items were used for participants who were trained with Set 1a and Set 1b items and vice versa (see Supplementary Material C).

#### Post-training materials

To form untrained items consisting of a trained first constituent and untrained second constituent (i.e., trained stem condition) and untrained first and second constituents (i.e., untrained stem condition) unlike in Experiment 1, each first constituent was combined with three different untrained second constituents. We used 12 second constituents and repeated each once. The same untrained second constituents were used in these conditions. The pictures, procedure, and analyses were the same as in Experiment 1.

### Results and discussion

#### Recognition task

##### Stem status

**Figure 4.**
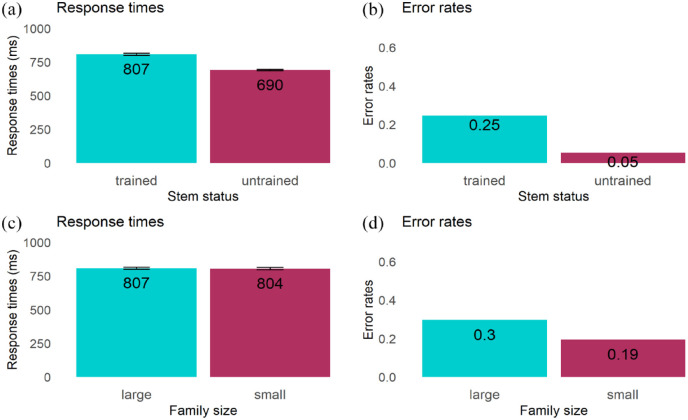
Experiment 2: Effect of stem status and family size in recognition task: (a) Mean response times for the effect of stem status, (b) Mean error rates (%) for the effect of stem status, (c) Mean response times for the effect of family size, and (d) Mean error rates (%) for the effect of family size..

*Response times.* We removed 716 errors (15%) out of 4,798 trials from the dataset. Trials above 2,500 ms were considered as outliers and removed (11 out of 4,082 correct responses). The remaining 4,071 trials were included in the analysis. The linear mixed-effects model with stem status as the fixed effect revealed a significant effect of stem status (β = 0.147, *SE* *=* 0.14, *t* = 10.32, *p* < .001), indicating slower responses to novel words containing a trained stem (*M*: 807 ms, *SD*: 302.5) than an untrained stem (*M*: 690 ms, *SD*: 226.4; see Figure 4a).

##### Error rates

The model revealed a significant effect of stem status (β = −2, *SE* *=* 0.217, *z* = −9.22, *p* < .001) reflecting participants’ less accurate responses in the trained stem condition (*M*: 24.5%, *SD*: 0.6) than in the untrained stem condition (*M*: 5%, *SD*: 0.31; see Figure 4b).

#### Morphological family size

##### Response times

We removed 589 errors (24.5%) out of 2,400 trials from the dataset. Outlier trials were also removed (7 out of 1,811 correct responses). The remaining 1,804 trials were included in the analysis. The mean response time was 807 ms (*SD* = 239) in the large stem family size condition and 804 ms (*SD* = 269) in the small stem family size condition. The results showed no significant effect of family size on response times (β = 0.013, *SE* = 0.013, *t* = 0.99, *p* = .327; see Figure 4c).

##### Error rates

The statistical model revealed a significant effect of family size (β = −0.70, *SE* = 0.167, *z* = -4.222, *p* < .001) indicating that participants made more errors rejecting items in the large (*M*: 29.6%, *SD*: 0.54) than in the small family size condition (*M*: 19.4%, *SD*: 0.46; see Figure 4d).

#### Spelling task

##### Stem status

The results showed a significant effect of stem status (β = 1.169, *SE* *=* 0.34, *z* = 3.436, *p* < .001). That is, participants produced more accurate spelling of the trained stems (*M*: 50.6%, *SD*: 0.66) than untrained stems (*M*: 67.8%, *SD*: 0.63).

##### Morphological family size

The mean spelling error rate was 50% (*SD* = 0.65) in the large stem family size condition and 51% (*SD* = 0.66) in the small stem family size condition. The statistical model showed no significant effect of family size on responses (β = 0.064, *SE* *=* 0.158, *z* = 0.405, *p* = .685).

In sum, the results of Experiment 2 largely replicated those of Experiment 1 within a more carefully counterbalanced experimental design. As opposed to Experiment 1, items with small and large morphological families were presented equally often within the post-training phase, thus suggesting that the observed family size effect was clearly due to the morphological family size manipulation in the training rather than post-training phase. It is worth noting that the RTs of Experiment 2 were generally shorter than in Experiment 1 (944 vs 777 ms). One potential explanation may be that participants in Experiment 1 were on average older (M = 30; *SD* = 9.7) than the participants in Experiment 2 (*M* = 25, *SD* = 9.1). However, participants’ reading fluency was comparable across experiments (Experiment 1, *M*: 205 vs Experiment 2, *M*: 202), and so the source of this difference remains unclear.

Finally, to account for the fact that some participants received more training exposures than others, based on the number of training repetitions each individual needed to reach the 90% accuracy threshold in the training phase, we conducted an additional, exploratory (not preregistered) set of analyses in which we added the number of training runs (performed by each participant separately to reach 90% accuracy) as a predictor. In Experiment 1, the number of training repetitions ranged from 3 to 9 repetitions, with a mean of 7.44 repetitions (*SD* = 2). In Experiment 2, the range was between 2 and 9 repetitions, with a mean of 7.57 repetitions (*SD*: 1.98). The analyses were conducted for both the recognition and spelling tasks and on response times and error rates for stem status and morphological family size. The results of both stem status and morphological family size showed that the addition of this factor did not change the direction or significance of our findings. The detailed analyses scripts and results are openly available in an Open Science Framework repository at https://osf.io/g827m/.

## General discussion

The present study used two novel word learning experiments to investigate the acquisition of morphologically complex words consisting of novel constituent morphemes (e.g., *torb* + *ilm* = *torblim*). During training, participants learned novel words by associating their written form with either of two pictures. The training was repeated until participants reached a 90% response accuracy. The training was completed relatively quickly (in 10–15 min; 7 training runs on average). Following training, participants performed a recognition task and a spelling task where the generalisability of their previously acquired morphemic knowledge was tested by comparing their responses with items composed of a trained first constituent and an untrained second constituent (e.g., *veam* + *elp* = *veamelp*) with items composed of two untrained constituents (e.g., *prish* + *ig* = *prishig*). In addition, we investigated if the effects of morpheme acquisition were modulated by differences in morphological family size.

Morphological processing was not a prerequisite to complete the current learning task. However, the findings of the recognition and spelling tasks revealed a robust effect of stem status, suggesting that instead of just learning whole words, participants were able to draw on their existing morphemic parsing skills to decompose the novel words into morphemic constituents during training and identify the trained units when embedded in untrained letter sequences, despite never being exposed to the constituents in isolation. The data of both experiments clearly support the hypothesis that participants went beyond the acquisition of whole letter strings and showed evidence of generalisation of the trained stems to a new morphemic context. The present findings highlight the salience of morphemic structure in novel word acquisition and suggest that the segmentation of orthographic input into units of meaning supports the development of new vocabulary.

Several decades of reading research have extensively investigated the processing of morphological structures during reading. Across a variety of experimental paradigms, and in particular masked prime lexical decision, it has been shown that skilled readers are experts at identifying morphemes from print (e.g., Grainger et al., 1991; Longtin et al., 2003; Rastle & Davis, 2008). Although there has been debate about whether the early stages of visual word recognition are based on a purely orthographic form of morphological analyses (“morpho-orthographic” processing; Beyersmann et al., 2016; Rastle et al., 2004) or whether semantics is essentially a part of morphological decomposition from the initial processing stages onwards (“morpho-semantic” processing; Feldman et al., 2009, 2015), prior findings suggest that skilled readers draw on their morphemic knowledge in highly rapid, automatic ways. The current study takes this work one step further and shows that readers can decipher the morphological structure of entirely novel letter strings and apply this newly acquired knowledge to other novel letter strings. Crucially, participants in our experiments did not have any prior knowledge of the embedded orthographic units and had to rely entirely on mapping letters (e.g., *torbilm*) onto meaning (“big ball”).

Across both experiments, the results of the recognition task evidenced an effect of morphological family size, showing that it was harder to correctly reject items containing embedded constituents with large morphological families, compared with items containing embedded constituents with small morphological families. Although the effect of morphological family size was only significant in the error but not in the response time data of the recognition task, the mean difference scores in response times and error rates went in the same direction. This pattern of results was robust and observed across both experiments. While the training involved linking novel letter strings with meaning (i.e., pictures), the post-training recognition task was specifically selected to tap into the more form-based segmentation mechanisms that are at play when processing complex words. The goal was to compare two conditions (large vs small family size condition) that were perfectly matched on the semantic and orthographic relationship between the whole item and its embedded constituents, as well as on the amount of exposure that participants received during training. The only aspect that differed between these two key conditions was their morphological family size. As such, it can be clearly ruled out that the observed findings were based on the recognition of orthographic form or meaning, thus highlighting the idea that the trained stems were processed as distinctly defined morphemic reading units.

In line with prior research suggesting that morphological family size generally tends to have a facilitatory effect on word recognition (e.g., Baayen et al., 1997; Bertram et al., 2000; Beyersmann & Grainger, 2018; De Jong et al., 2002; Moscoso del Prado Martin et al., 2004), we are reporting that novel words with large morphological families are more generalisable than novel words with small morphological families. This result aligns with Tamminen et al.’s (2015) prior findings showing that words with a large morphological family are learned better than words with a small morphological family. The here observed effect of morphological family size also fits with the notion that “word families” can be beneficial in the development of reading skills (e.g., Nation, 2021). Nation proposes that learning is clustered around “base words” in the form of word families, allowing learners to more easily recognise and understand new words in texts. For example, if a learner knows the base word “quantify” and its related words “quantitative,” and “quantifier,” they will find it easier to recognise “quantifiable” when encountered during reading. Accordingly, Nation (2013) emphasises that teaching vocabulary in families can provide learners with a deeper understanding of the meaning of words and lead to better recall, which is consistent with the current findings.

## Limitations and future directions

While the reported facilitatory effect of morphological family size was observed across both experiments and also in line with Tamminen et al.’s (2015) earlier findings, it cannot be ruled out from the current post-training data that the effect of morphological family size may be reversed during the learning process itself. It may indeed be the case that the learning process is impeded for words with large morphological families based on the negative effect of context variability for items in this category, as would be predicted by a number of recent distributional learning theories (e.g., Baayen et al., 2011; Raviv et al., 2022). This is quite the opposite of the here reported facilitatory effect of morphological family size on post-training, suggesting that while morphological family size seems to facilitate mechanisms of generalisation, it may indeed have an inhibitory effect on training. This line of research requires further investigation, using fine-tuned methods that are better able to track the mechanisms involved in the dynamics of the learning process itself.

It is also worth noting that the present study used novel items that were orthographically and phonologically regular, thus assisting participants’ learning of the novel morphemes. The role of orthographic and phonological regularity in learning words with multiple morphemes may be addressed by systematically varying the degree of orthographic and phonological consistency across item types. Another promising future extension of the current findings may be to use the current experimental paradigm to test the acquisition of novel morphemic knowledge by training merely the orthographic form of novel words and without explicit semantic instruction through picture–word associations. In the current study, the training involved teaching participants form–meaning associations, and as such we did not test the situation where morphological structure was taught without semantics. Directly comparing complex novel word training with and without semantics would inform the question of whether complex word learning can be based on a more abstract, semantically independent form of morphological analysis.

Finally, another important extension of the current work is the investigation of complex novel word acquisition in developing readers. We assume that the kind of learning principles that we observed in our present sample of skilled readers, also apply to children who are still in the process of learning to read (Raviv et al., 2022). The current novel word learning paradigm mimics a number of key principles that are fundamental to the acquisition of complex words in young readers. Beginning readers use morphological analysis as a tool to “infer the meaning of unfamiliar morphologically complex words on the spot based on their morpheme constituents” (Levesque et al., 2019, p. 64). Crucially, children already have knowledge of semantic morphemic representations prior to learning to read (Beyersmann, Wegener, Spencer, & Castles, 2022), based on form–meaning regularities they have been exposed to in their spoken language acquisition (Beyersmann & Grainger, 2023). English-speaking children as young as third graders can interpret the meaning of morphologically complex words (Anglin et al., 1993; Crosson & McKeown, 2016; McCutchen & Logan, 2011; Ram et al., 2013; Tyler & Nagy, 1989; Wysocki & Jenkins, 1987). However, once children begin to learn to read, it takes a substantial amount of time to establish connections between orthographic input and semantics (e.g., Beyersmann et al., 2012; for related evidence from L2 speakers see Menut et al., 2023) presumably based on the successful phonological decoding of unfamiliar orthographic stimuli (e.g., Grainger et al., 2012; Share, 1995; Ziegler et al., 2014). If it turns out to be true that words containing morphemes with a larger family size are easier to read than words with a smaller family size, this information can be used when planning instruction to enhance literacy skills.

## Conclusion

The present study used a novel word training paradigm to examine the acquisition of morphologically complex novel words consisting of two novel morphemes. The results of two training experiments provided evidence that participants were able to identify the embedded morphemic constituents without the support of any prior morphological and lexical knowledge and without ever previously seeing the embedded constituents in isolation. Moreover, the findings shed light on the facilitatory impact of morphological family size, suggesting that morphological knowledge expedites the process of novel word acquisition.

## Supplemental Material

sj-docx-1-qjp-10.1177_17470218231216369 – Supplemental material for The role of morphemic knowledge during novel word learning Supplemental material, sj-docx-1-qjp-10.1177_17470218231216369 for The role of morphemic knowledge during novel word learning by Ali Behzadnia, Johannes C. Ziegler, Danielle Colenbrander, Audrey Bürki and Elisabeth Beyersmann in Quarterly Journal of Experimental Psychology

## Notes

1. To account for the fact that some participants received more training exposures than others, based on the number of training repetitions each individual needed to reach the 90% accuracy threshold in the training phase, we conducted an additional, more exploratory set of analyses in which we added the number of training runs (performed by each participant separately to reach 90% accuracy) as a predictor. The analyses were conducted for both the recognition and the spelling tasks and on response times and error rates for stem status and morphological family size. The results of both stem status and morphological family size showed that the addition of this factor did not change the direction or significance of our findings. The analysis scripts and results are openly available in [Open Science Framework] at https://osf.io/g827m/.
