# Supplementary material for: The role of morphemic knowledge during novel word learning
Source: Q J Exp Psychol (Hove). 2023 Dec 7;77(8):1620–34. doi: 10.1177/17470218231216369 (PMC11295409; doi:10.1177/17470218231216369)
Supplement: sj-docx-1-qjp-10.1177_17470218231216369 – Supplemental material for The role of morphemic knowledge during novel word learning [file sj-docx-1-qjp-10.1177_17470218231216369.docx]

Supplementary Material for:

**The Role of Morphemic Knowledge during Novel Word Learning**

Ali Behzadnia ^a, b, c, d^, Johannes Ziegler ^e^, Danielle Colenbrander ^c, d, f^, Audrey Bürki^* a^, and Elisabeth Beyersmann^* c, d^

^a^ Department of Linguistics, University of Potsdam

^b^ International Doctorate for Experimental Approaches to Language and Brain (IDEALAB): Universities of Potsdam (DE), Groningen (NL), Newcastle (UK), and Macquarie University (AU).

^c^ School of Psychological Sciences, Macquarie University, Sydney, New South Wales 2109, Australia

^d^ Macquarie University Centre for Reading, Macquarie University, Sydney, New South Wales 2109, Australia

^e^ Aix-Marseille Université, Centre National de la Recherche Scientifique, Laboratoire de Psychologie Cognitive, Marseille, France

^f^ Australian Centre for the Advancement of Literacy (ACAL), Faculty of Education and Arts, Australian Catholic University, Sydney, Australia

***** Audrey Bürki and Elisabeth Beyersmann should be considered joint senior authors.

Correspondence concerning this article should be addressed to Ali Behzadnia, School of Psychological Sciences and Macquarie University Centre for Reading (MQCR), University of Macquarie, Level 3, Australian Hearing Hub, 16 University Avenue, Sydney, Australia. Email: [ali.behzadnia@hdr.mq.edu.au](mailto:ali.behzadnia@hdr.mq.edu.au)

**Supplementary Material A**

**List of the novel words and their meanings in Experiment 1**

|  | Set 1 | | | Set 2 | | |
| --- | --- | --- | --- | --- | --- | --- |
| Condition | First constituent + second constituent | Whole word | Meaning | First constituent + second constituent | Whole word | Meaning |
| Large family size | farsh + erp | farsherp | red fish | greach + el | greachel | red fish |
|  | farsh + lor | farshlor | blue fish | greach + ilt | greachilt | blue fish |
|  | farsh + oth | farshoth | striped fish | greach + rup | greachrup | striped fish |
|  | farsh + ib | farshib | polka dot fish | greach + isp | greachisp | polka dot fish |
|  | kirth + ift | kirthift | cheap car | keaf + lem | keaflem | cheap car |
|  | kirth + iom | kirthiom | expensive car | keaf + elp | keafelp | expensive car |
|  | kirth + erp | kirtherp | red car | keaf + el | keafel | red car |
|  | kirth + lor | kirthlor | blue car | keaf + ilt | keafilt | blue car |
|  | breap + ord | breapord | clean t-shirt | haith + asm | haithasm | clean t-shirt |
|  | breap + nel | breapnel | dirty t-shirt | haith + ig | haithig | dirty t-shirt |
|  | breap + oth | breapoth | striped t-shirt | haith + rup | haithrup | striped t-shirt |
|  | breap + ib | breapib | polka dot t-shirt | haith + isp | haithisp | polka dot t-shirt |
|  | veam + ift | veamift | cheap bag | prish + lem | prishlem | cheap bag |
|  | veam + iom | veamiom | expensive bag | prish + elp | prishelp | expensive bag |
|  | veam + ord | veamord | clean bag | prish + asm | prishasm | clean bag |
|  | veam + nel | veamnel | dirty bag | prish + ig | prishig | dirty bag |
| Small family size | dirch + ilm | dirchilm | big coin | smeel + po | smeelpo | big coin |
|  | dirch + la | dirchla | small coin | smeel + erv | smeelerv | small coin |
|  | spoal + iph | spoaliph | old bike | prail + urt | prailurt | old bike |
|  | spoal + ret | spoalret | new bike | prail + afe | prailafe | new bike |
|  | torb + ilm | torbilm | big ball | bloam + po | bloampo | big ball |
|  | torb + la | torbla | small ball | bloam + erv | bloamerv | small ball |
|  | shoom + iph | shoomiph | old hat | voop + urt | voopurt | old hat |
|  | shoom + ret | shoomret | new hat | voop + afe | voopafe | new hat |

**Supplementary Material B**

**List of the novel words and their meanings in Experiment 2**

|  | Set 1a | | | Set 1b | | |
| --- | --- | --- | --- | --- | --- | --- |
| Condition | First constituent + second constituent | Whole word | Meaning | First constituent + second constituent | Whole word | Meaning |
| Large family size | farsh + erp | farsherp | red fish | dirch + erp | dircherp | red fish |
|  | farsh + lor | farshlor | blue fish | dirch + iph | dirchiph | blue fish |
|  | farsh + oth | farshoth | striped fish | dirch + ift | dirchift | striped fish |
|  | farsh + ib | farshib | polka dot fish | dirch + iom | dirchiom | polka dot fish |
|  | kirth + ift | kirthift | cheap car | spoal + ib | spoalib | cheap car |
|  | kirth + iom | kirthiom | expensive car | spoal + iom | spoaliom | expensive car |
|  | kirth + erp | kirtherp | red car | spoal + erp | spoalerp | red car |
|  | kirth + lor | kirthlor | blue car | spoal + nel | spoalnel | blue car |
|  | breap + ord | breapord | clean t-shirt | torb + ilm | torbilm | clean t-shirt |
|  | breap + nel | breapnel | dirty t-shirt | torb + la | torbla | dirty t-shirt |
|  | breap + oth | breapoth | striped t-shirt | torb + iph | torbiph | striped t-shirt |
|  | breap + ib | breapib | polka dot  t-shirt | torb + nel | torbnel | polka dot t-shirt |
|  | veam + ift | veamift | cheap bag | shoom + ilm | shoomilm | cheap bag |
|  | veam + iom | veamiom | expensive bag | shoom + la | shoomla | expensive bag |
|  | veam + ord | veamord | clean bag | shoom + ib | shoomib | clean bag |
|  | veam + nel | veamnel | dirty bag | shoom + ift | shoomift | dirty bag |
| Small family size | dirch + ilm | dirchilm | big coin | farsh + ord | farshord | big coin |
|  | dirch + la | dirchla | small coin | farsh + ret | farshret | small coin |
|  | spoal + iph | spoaliph | old bike | kirth + oth | kirthoth | old bike |
|  | spoal + ret | spoalret | new bike | kirth + ord | kirthord | new bike |
|  | torb + ilm | torbilm | big ball | breap + ret | breapret | big ball |
|  | torb + la | torbla | small ball | breap + lor | breaplor | small ball |
|  | shoom + iph | shoomiph | old hat | veam + oth | veamoth | old hat |
|  | shoom + ret | shoomret | new hat | veam + lor | veamlor | new hat |

|  | Set 2a | | | Set 2b | | |
| --- | --- | --- | --- | --- | --- | --- |
| Condition | First constituent + second constituent | Whole word | Meaning | First constituent + second constituent | Whole word | Meaning |
| Large family size | greach + el | greachel | red fish | smeel + ig | smeelig | red fish |
|  | greach + ilt | greachilt | blue fish | smeel + asm | smeelasm | blue fish |
|  | greach + rup | greachrup | striped fish | smeel + afe | smeelafe | striped fish |
|  | greach + isp | greachisp | polka dot fish | smeel + rup | smeelafe | polka dot fish |
|  | keaf + lem | keaflem | cheap car | prail + asm | prailasm | cheap car |
|  | keaf + elp | keafelp | expensive car | prail + ilt | praililt | expensive car |
|  | keaf + el | keafel | red car | prail + rup | prailrup | red car |
|  | keaf + ilt | keafilt | blue car | prail + isp | prailisp | blue car |
|  | haith + asm | haithasm | clean t-shirt | bloam + afe | bloamafe | clean t-shirt |
|  | haith + ig | haithig | dirty t-shirt | bloam + elp | bloamelp | dirty t-shirt |
|  | haith + rup | haithrup | striped t-shirt | bloam + ig | bloamig | striped t-shirt |
|  | haith + isp | haithisp | polka dot  t-shirt | bloam + el | bloamel | polka dot t-shirt |
|  | prish + lem | prishlem | cheap bag | voop + isp | voopsip | cheap bag |
|  | prish + elp | prishelp | expensive bag | voop + ilt | voopilt | expensive bag |
|  | prish + asm | prishasm | clean bag | voop + elp | voopelp | clean bag |
|  | prish + ig | prishig | dirty bag | voop + el | voopel | dirty bag |
| Small family size | smeel + po | smeelpo | big coin | greach + lem | greachlem | big coin |
|  | smeel + erv | smeelerv | small coin | greach + po | greachpo | small coin |
|  | prail + urt | prailurt | old bike | keaf + urt | keafurt | old bike |
|  | prail + afe | prailafe | new bike | keaf + erv | keaferv | new bike |
|  | bloam + po | bloampo | big ball | haith + urt | haithurt | big ball |
|  | bloam + erv | bloamerv | small ball | haith + lem | haithlem | small ball |
|  | voop + urt | voopurt | old hat | prish + erv | prisherv | old hat |
|  | voop + afe | voopafe | new hat | prish + po | prishpo | new hat |

**Supplementary Material C**

**List of the items in the post-training phase in Experiment 2**

| Set1a | | | Set1b | | |
| --- | --- | --- | --- | --- | --- |
| Condition | | | Condition | | |
| Trained item | Trained stem | Untrained stem | Trained item | Untrained stem | Untrained stem |
| farsherp | farshilt | greachel | dircherp | dirchurt | greachel |
| farshlor | farshig | greachilt | dirchiph | dirchpo | greachilt |
| farshoth | farshlem | greachrup | dirchift | dirchilt | greachrup |
| farshib | kirthrup | keafelp | dirchiom | spoalerv | keafelp |
| kirthift | kirthel | keafilt | spoalib | spoalafe | keafilt |
| kirthiom | kirthig | keafrup | spoaliom | spoalisp | keafrup |
| kirtherp | breapisp | haithasm | spoalerp | torburt | haithasm |
| kirthlor | breapasm | haithig | spoalnel | torbpo | haithig |
| breapord | breaplem | haithisp | torbilm | torbelp | haithisp |
| breapnel | veamrup | prishelp | torbla | shoomerv | prishelp |
| breapoth | veamel | prishlem | torbiph | shoomafe | prishlem |
| breapib | veamasm | prishig | torbnel | shoomelp | prishig |
| veamift | dirchurt | smeelpo | shoomilm | farshilt | smeelpo |
| veamiom | dirchpo | smeelerv | shoomla | farshig | smeelerv |
| veamord | dirchilt | smeelafe | shoomib | farshlem | smeelafe |
| veamnel | spoalerv | prailurt | shoomift | kirthrup | prailurt |
| dirchilm | spoalafe | prailafe | farshord | kirthel | prailafe |
| dirchla | spoalisp | prailasm | farshret | kirthig | prailasm |
| spoaliph | torburt | bloamerv | kirthoth | breapisp | bloamerv |
| spoalret | torbpo | bloampo | kirthord | breapasm | bloampo |
| torbilm | torbelp | bloamisp | breapret | breaplem | bloamisp |
| torbla | shoomerv | voopurt | breaplor | veamrup | voopurt |
| shoomiph | shoomafe | vooplem | veamoth | veamel | vooplem |
| shoomret | shoomelp | voopel | veamlor | veamasm | voopel |

| Set2a | | | Set2b | | |
| --- | --- | --- | --- | --- | --- |
| Condition | | | Condition | | |
| Trained item | Trained stem | Untrained stem | Trained item | Untrained stem | Untrained stem |
| greachel | greachla | farshlor | smeelig | smeeliph | farshlor |
| greachilt | greachlor | farshoth | smeelasm | smeelib | farshoth |
| greachrup | greachilm | farshnel | smeelafe | smeeliom | farshnel |
| greachisp | keafiom | kirtherp | smeelrup | prailerp | kirtherp |
| keaflem | keafla | kirthlor | prailasm | prailiph | kirthlor |
| keafelp | keafoth | kirthiom | praililt | prailret | kirthiom |
| keafel | haithlor | breapoth | prailrup | bloamret | breapoth |
| keafilt | haithib | breapnel | prailisp | bloamord | breapnel |
| haithasm | haithilm | breapib | bloamafe | bloamift | breapib |
| haithig | prisherp | veamord | bloamelp | voopift | veamord |
| haithrup | prishoth | veamiom | bloamig | voopnel | veamiom |
| haithisp | prishnel | veamift | bloamel | voopord | veamift |
| prishlem | smeeliph | dirchilm | voopsip | greachla | dirchilm |
| prishelp | smeelib | dirchla | voopilt | greachlor | dirchla |
| prishasm | smeeliom | dirchift | voopelp | greachilm | dirchift |
| prishig | prailerp | spoaliph | voopel | keafiom | spoaliph |
| smeelpo | prailiph | spoalib | greachlem | keafla | spoalib |
| smeelerv | prailret | spoalerp | greachpo | keafoth | spoalerp |
| prailurt | bloamret | torbret | keafurt | haithlor | torbret |
| prailafe | bloamord | torbilm | keaferv | haithib | torbilm |
| bloampo | bloamift | torbla | haithurt | haithilm | torbla |
| bloamerv | voopift | shoomiph | haithlem | prisherp | shoomiph |
| voopurt | voopnel | shoomret | prisherv | prishoth | shoomret |
| voopafe | voopord | shoomord | prishpo | prishnel | shoomord |
